# Obesity-associated hypertension is ameliorated in patients with TLR4 single nucleotide polymorphism (SNP) rs4986790

**DOI:** 10.1186/s12950-015-0100-5

**Published:** 2015-10-01

**Authors:** Simon Schneider, Petra Hoppmann, Werner Koch, Stephan Kemmner, Christoph Schmaderer, Lutz Renders, Adnan Kastrati, Karl-Ludwig Laugwitz, Uwe Heemann, Marcus Baumann

**Affiliations:** Department of Nephrology, Klinikum rechts der Isar, Technical University Munich, Ismaninger St. 22, 81675 Munich, Germany; I. Medizinische Klinik und Poliklinik, Klinikum rechts der Isar, Technical University Munich, Munich, Germany; Deutsches Herzzentrum München, Technical University Munich, Munich, Germany; German Centre for Cardiovascular Research (DZHK), partner site Munich Heart Alliance, Munich, Germany

**Keywords:** Obesity associated hypertension, Systolic blood pressure, Toll-like receptor 4, TLR4, Genetics

## Abstract

**Background:**

Obesity is strongly associated with hypertension. Despite numerous mechanistic links the association is not fully understood. Western diet increases uptake of Toll-Like receptor 4 (TLR4) ligands such as free fatty acids or endotoxin. We recently demonstrated that TLR4 ligands are involved in the development of hypertension. We hypothesized that TLR4 ligands are involved in obesity-associated hypertension and investigated the TLR4 single nucleotide polymorphism (SNP rs 498790). This SNP is frequent, associated with cardiovascular disease and characterized by blunted response upon exposure to TLR4 ligands.

**Methods:**

We investigated 3657 patients undergoing coronary angiography. Blood pressure was determined in standardized manner prior angiography. The diagnosis of hypertension was based on record data. Patients were characterized for TLR4 single nucleotide polymorphism (SNP) rs4986790. Patients were stratified according to quartiles of Body mass index (BMI) and according to the polymorphism. The association between the TLR4 polymorphism and blood pressure in obese patients (BMI > 30 kg/m^2^) was investigated by multivariate regression analysis.

**Results:**

Out of 3657 patients 3017 patients fulfilled inclusion criteria. In the whole cohort a significant increase of SBP, pulse pressure and diagnosis of hypertension was observed across BMI quartiles. By contrast, no significant increase of SBP, pulse pressure or diagnosis of hypertension was observed in the 319 cases with TLR4 SNP rs4986790 across BMI quartiles. These obese cases had significantly lower SBP, lower pulse pressure (7.0 and 7.6 mmHg) and less diagnosis of hypertension as controls. In obesity the TLR4 SNP rs4986790 was an independent predictor of SBP.

**Conclusion:**

Systolic blood pressure increase with obesity was blunted in cases with TLR4 SNP rs4986790.

## Background

The increasing prevalence of obesity worldwide is a serious health hazard. Obese individuals are at increased risk for diabetes, hypertension, renal failure, and other cardiovascular diseases. Clinical studies have confirmed a strong relationship between obesity and hypertension [1, 2]. In large epidemiological studies the increased risk for cardiovascular diseases attributed to overweight requires the presence of arterial hypertension, a key risk factor for most of cardiovascular diseases [3]. Excess weight is clearly related to increase in blood pressure levels [4]. However, despite a number of therapeutic options available, adequate blood pressure control is achieved only in half of these patients [5], evidencing that the mechanisms involved in blood pressure increase associated to obesity still need to be better understood [6, 7].

Chronic low-grade inflammation is linked to hypertension, being one important feature shared by obesity and hypertension [8, 9]. This link has been poorly explored, although several sources for low-grade inflammation in obesity are known. Diet-induced obesity is associated with ligands of the Toll-like receptor (TLR) 4, an evolutionarily ancient pattern recognition receptor that plays a key role in the innate immune response. Western diet works as an important trigger of TLR4 signaling, as it provides ligands such as free fatty acids [10, 11]. Another source of TLR4 activation is the translocation of bacterial endotoxin [lipopolysaccharide (LPS)] from the gut into the blood stream [12], the so-called metabolic endotoxemia that occurs with diet-induced obesity [13]. Several TLR 4 SNPs have been described. We were interested in a TLR4 SNP which is frequent and has a reduced reagibility upon TLR4 ligands as we hypothesize that in this case it would be associated with reduced blood pressure. These factors are fulfilled by the TLR4 SNP rs4986790. This SNP showed a reduced reagiblity on LPS [14]. Moreover this SNP belongs to the most frequent TLR 4 SNPs [15]. Furthermore we could recently find an association of this TLR 4 SNP rs 4986790 with age-dependant blood pressure increase in patients with coronary artery disease [16]. Therefore we investigated whether cases with a TLR4 SNP rs4986790 had ameliorated blood pressure in obesity as compared to controls.

## Methods

### Patients

Participants were examined at I. Medizinische Klinik rechts der Isar der Technischen Universität München or Deutsches Herzzentrum München. Between 1993 and 2002, 3657 patients were enrolled in a dedicated registry including patients with and without myocardial infarction [15]. The local ethics committee approved the registry. A consent to participate and to publish was obtained. Patients with cardiogenic shock and advanced heart insufficiency defined as a SBP < 100 mmHg were excluded, finally reaching a cohort size of 3017 patients.

SBP and DBP were determined under standardized conditions in the laying position (Cardis, Schwarzer GmbH). Clinical and laboratory data were prospectively collected in an online database. Source data check was done with 20 % of the data. We selected from the TLR4 genes 12 reported SNPs and focused on the rs4986790 (Asp299Gly) as this SNP is frequently present and this SNP is known to demonstrate reduced activity on TLR4 ligands.

In summary, a blood sample was drawn from each participant during the regular routine examination for coronary artery angiogram. Genomic DNA was extracted from peripheral blood leukocytes with the QIAamp DNA Blood Kit (Qiagen, Hilden, Germany) or the High Pure PCR Template Preparation Kit (Roche Applied Science, Mannheim, Germany). Genotype analysis was performed with allele-specific fluorogenic oligonucleotide probes in an assay combining the polymerase chain reaction (PCR) and the 5′ nuclease reaction (TaqMan technique; Applied Biosystems, Darmstadt, Germany). Primers and probes were established with the Primer Express software (version 2.0.0; Applied Biosystems) after import of a DNA sequence (Homo sapiens *TLR4* gene, TLR4A allele) deposited under accession number AF177765 in GenBank (http://www.ncbi.nlm.nih/entrez/). The primers used for amplification of the SNP rs4986790 (Asp299Gly) were 5′ TTGAAGAATTCCGATTAGCATACTTAGAC 3′ and 5′ TCACCAGGGAAAATGAAGAAACAT 3′. Allele-specific signalling was accomplished with the use of the fluorogenic dyes 6-carboxy-fluorescein (FAM) and VIC (proprietary dye of Applied Biosystems) which were attached to the 5′ end of the probe oligonucleotides. Minor groove binder (MGB) groups were conjugated with the 3′ end of the oligonucleotides to facilitate formation of stable duplexes between the probes and their single-stranded DNA targets. The structures of the probes were as follows (allele-specific nucleotides are underlined): FAM-5′ CCTCGATGATATTATTGACT 3′-MGB (for 896A), VIC-5′ CTCGATGGTATTATTG 3′-MGB (for 896G).[17] Oligonucleotides were synthesized by Applied Biosystems. The two-step thermocycling procedure consisted of 35 cycles of denaturation at 92 °C for 15 s and primer annealing and extension at 60 °C for 1 min. After cycling on a GeneAmp PCR System 9600 or 9700 (Applied Biosystems), endpoint fluorescent data acquisition and genotype calling was achieved on an ABI Prism 7000 Sequence Detection System (Applied Biosystems). As a control, genotyping was repeated for 20 % of the samples with the use of DNA prepared separately from the original blood sample. The ability of the TaqMan systems to provide correct genotype data was verified in separate analyses of 200 different PCR products with the allele-discriminating restriction enzyme *Bcc*I (New England Biolabs, Frankfurt am Main, Germany) (TLR4 SNP rs4986790). The results obtained with the different methods were fully consistent, which demonstrated the reliability of the TaqMan systems that were used for genotyping of the SNP rs4986790 in the entire study population. Genotype determination was done by workers who had no knowledge of clinical, laboratory, or angiographic data of the individuals enrolled in the study.

### Data analysis and statistical procedures

Data are presented as mean ± SD. The analysis compaired genotype distributions between the BMI quartiles. The BMI quartile ranges for the whole cohort were for quartile 1 (Q1) a BMI < 24,64, for quartile 2 (Q2) BMI ≥24.65-26,89, for quartile 3 (Q3) BMI ≥ 26.90-29.50 and for quartile 4(Q4) BMI ≥ 29,51 kg/m^2^. The primary model was to assess the relation between blood pressure across BMI quartiles in cases and controls. In the next step we focused on the upper BMI quartile and compared blood pressure between cases and controls. Discrete variables are expressed as counts (percentage) and compared with the use of the *χ*^2^ test or Fisher’s exact test, as appropriate. Continuous variables are compared by means of the unpaired, two-sided *t* test. Genotype frequencies in the case group were compared with values predicted by Hardy–Weinberg equilibrium with the use of the *χ*^2^ test. Multivariate analysis used age, gender and hypercholesterolemia. Statistical significance was accepted for *P*-values <0.05. The probability of a false negative association result because of population stratification is low, because the study participants were consecutively recruited from a defined geographic area of southern Germany with limited recent immigration. The sample size allowed an analysis with 80 % power to detect a six mmHg decrease of SBP among the cases with the TLR4 SNP rs4986790 at a two-sided **α**-error of 0.05.

## Results

We investigated a cohort of 3017 patients who underwent coronary angiography. Distribution of the patients according to quartiles of body mass index (BMI) revealed no differences for age, gender or smoking. However the metabolic profile of the patients in the upper BMI quartile was markedly altered. Lipid profile showed higher triglycerides and lower HDL-cholesterol in the upper BMI quartile. In the upper quartile the diagnosis of diabetes and hypercholesterolemia was higher as compared to other BMI quartiles.

Similarly systolic blood pressure, pulse pressure and diagnosis of hypertension increased across BMI quartiles whereas diastolic blood pressure remained stable. In line with the presence of hypertension in the upper BMI quartile the number of antihypertensive drugs was higher in the upper quartile (Table 1). The same findings were present if analysis was restricted to controls.

**Table 1:** Patient characteristics of the whole study population devided by BMI quartiles

	Whole Cohort (*n* = 3017)			
BMI, quartiles	Q1	Q2	Q3	Q4
Number, *n* (%)	754 (25)	756 (25)	752 (25)	755 (25)
Age, y	56,6 ± 9,5	57,6 ± 9,1	57,0 ± 8,5	57,1 ± 8,9
Sex, female,*n* (%)	272 (36)	142 (19)	158 (21)	209 (28)
BMI, kg/m^2^	22,6 ± 1,7	25,8 ± 0.7	28,1 ± 0.8	32,7 ± 3,1*
Smoking, *n* (%)	344 (46)	355 (47)	362 (48)	345 (46)
Diabetes, *n* (%)	90 (12)	93 (12)	85 (11)	145 (19)*
Hypercholesterolemia, *n* (%)	387 (51)	444 (59)	420 (56)	450 (60)*
LDL, mg/dl	122,6 ± 39,9	126,3 ± 40,3	125,5 ± 41,3	122,4 ± 40,5
HDL, mg/dl	55,3 ± 31,8	49,5 ± 16,0	46,9 ± 15,6	45,3 ± 13,0*
TG, mg/dl	134,8 ± 132,1	157,6 ± 111,2	169,8 ± 106.4	198,2 ± 192,1*
SBP, mmHg	144,5 ± 26,8	144,9 ± 25,6	145,2 ± 25,4	147,8 ± 26,2*
DBP, mmHg	79,5 ± 3,6	79,6 ± 3,7	79,5 ± 3,8	79,7 ± 3,6
PP, mmHg	64,7 ± 26,8	65,6 ± 25,4	65,8 ± 24,9	68,1 ± 26,2*
Hypertension, n (%)	401 (53)	429 (57)	426 (57)	(454) 60*
Antihypertensive drugs, n	1,14 ± 1.07	1,26 ± 1,07	1,31 ± 1,06	1,46 ± 1,09*
HR, bpm	75,3 ± 12,5	73,5 ± 12,6	73,7 ± 12,3	73,6 ± 12,6
MI, *n* (%)	494 (66)	561 (74)	548 (73)	530 (70)

Data are presented as mean ± SD; other variables are presented as number (%), Q1-Q4: quartile 1-4, BMI quartile ranges: Q1: BMI < 24,64, Q2: BMI ≥24.65-26,89, Q3: BMI ≥ 26. 26.90-29.50, Q4: BMI ≥ 29,51; *BMI* body mass index, *LDL* low-density lipoprotein, *HDL* high-density lipoprotein, *TG* triglycerides, *SBP* systolic blood pressure, *DBP* diastolic blood pressure, *PP* pulse pressure, *HR* heart rate, *MI* myocardial infarction; *P for trend <0.05

In this cohort, 319 cases with the TLR4 SNP rs4986790 were identified. The TLR4 SNP rs4986790 cases were distributed according to the Hardy-Weinberg equilibrium (HWE = 0.32). In comparison to controls, TLR4 SNP rs4986790 cases were comparable for age, gender and smoking habits (Table 2). Similarly to controls triglycerides increased and HDL-cholesterol reduced with BMI. No effect was seen for LDL-cholesterol. The diagnosis of diabetes and hypercholesterolemia across BMI quartiles showed a tendency of 15 vs. 20 % and 56 vs. 60 % (cases vs. controls) in the highest quartile, but no significance. In contrast to controls the TLR4 SNP rs4986790 cases did not demonstrate the blood pressure increase across the BMI quartiles despite comparable general characteristics. In the upper BMI quartile the cases with the TLR4 SNP rs4986790 the systolic blood pressure was 7 mmHg lower as compared to controls. Antihypertensive treatment did not explain the blood pressure difference between the cohorts.

**Table 2:** Comparison of controls and TLR4 SNP rs4986790 cases divided by BMI Quartiles

	Controls (*n* = 2698)				Cases (*n* = 319)			
BMI, quartiles	Q1	Q2	Q3	Q4	Q1	Q2	Q3	Q4
Number, *n* (%)	665/754 (88,2)	674/756 (89,2)	676/752 (89,9)	683/755 (90,5)	89/754 (11,8)	82/756 (10,8)	76/752 (10,1)	72/755 (9,5)
Age, y	56,7 ± 9,4	57,4 ± 9,2	57,0 ± 8,6	57,0 ± 8,9	55,2 ± 10,1	58,7 ± 8,0	57,3 8,1	57,6 ± 9,2
Sex, female *n* (%)	239 (36)	128 (19)	135 (20)	191 (28)	33 (37)	14 (17)	23 (30)	18 (25)
BMI, kg/m^2^	22,6 ± 1,7	25,8 ± 0,7	28,2 ± 0,8	32,7 ± 3,0*	22,6 ± 1,7	25,8 ± 0,6	28,1 ± 0,7	33,0 ± 3,3*
Smoking, *n* (%)	300 (45)	317 (47)	331 (49)	314 (46)	44 (49)	38 (46)	31 (41)	31 (43)
Diabetes, *n* (%)	80 (12)	81 (12)	68 (10)	134 (20)*	10 (11)	12 (15)	17 (22)	11 (15)
Cholesterolemia, n (%)	337 (51)	393 (58)	379 (56)	410 (60)*	50 (56)	51 (62)	41 (54)	40 (56)
LDL, mg/dl	122,1 ± 40,0	126,5 ± 40,8	126,5 ± 41,8	122,9 ± 41,5	126,7 ± 39,3	124,7 ± 36,8	116,2 ± 36,5	118,1 ± 30,2
HDL, mg/dl	55,3 ± 33,4	49,7 ± 16,3	47,2 ± 16,0	45,4 ± 13,2*	54,4 ± 16,0	48,1 ± 13,5	44,2 ± 11,7	44,6 ± 10,7*
TG, mg/dl	137,3 ± 139,0	154,4 ± 111,7	171,0 ± 104,5	198,1 ± 195,8*	116,7 ± 62,2	184,0 ± 105,0	158,6 ± 121,9	198,9 ± 156,7*
SBP, mmHg	144,7 ± 26,6	144,4 ± 25,5	145,2 ± 25,4	148,5 ± 26,3*	143,3 ± 28,1	146,6 ± 23,6	144,4 ± 26,0	141,5 ± 24,2^**#**^
DBP, mmHg	79,4 ± 3,6	79,7 ± 3,8	79,6 ± 3,6	79,8 ± 3,5	80,0 ± 3,9	79,6 ± 2,6	79,3 ± 5,4	79,1 ± 4,6
PP, mmHg	64,9 ± 25,6	65,2 ± 25,3	65,8 ± 25,8	68,8 ± 26,1*	62,8 ± 27,8	66,8 ± 24,6	65,7 ± 25,6	61,2 ± 22,8^**#**^
Hypertension, n (%)	359 (54)	380 (56)	386 (57)	417 (61)*	42 (47)	49 (60)	40 (53)	37 (51)
Antihypertensive drugs, n	1,15 ± 1,08	1,24 ± 1,08	1,31 ± 1,07	1,48 ± 1,10*	1,05 ± 1,01	1,32 ± 1,00	1,32 ± 0.97	1,23 ± 1,04
HR, bpm	75,2 ± 12,5	73,4 ± 12,7	73,6 ± 12,7	73,7 ± 12,6	75,3 ± 12,5	74,2 ± 11,6	75,3 ± 11,6	72,8 ± 12,3
MI, %	441 (66)	504 (75)	493 (73)	477 (70)	53 (60)	57 (70)	55 (72)	53 (74)

Data are presented as mean ± SD; other variables are presented as number (%), Q1-Q4: quartile 1-4, BMI quartile ranges: Q1: BMI < 24,64, Q2: BMI ≥24.65-26,89, Q3: BMI ≥ 26. 26.90-29.50, Q4: BMI ≥ 29,51, *BMI* body mass index, *LDL* low-density lipoprotein, *HDL* high-density lipoprotein, *TG* triglycerides, *SBP* systolic blood pressure, *DBP* diastolic blood pressure, *PP* pulse pressure, *HR* heart rate, *MI* myocardial infarction, *P for trend <0.05, ^#^ *P* < 0.05 controls versus TLR4 SNP rs4986790 cases

To assess the independent contribution of the TLR4 SNP rs4986790 on blood pressure in obese patients, we performed a multivariate regression in patients with a BMI ≥ 30 kg/m^2^ (Table 3). The TLR4 SNP rs4986790 remained a significant factor predicting blood pressure in obese patients despite multiple adjustments.

**Table 3:** Multivariate regression analysis of obese patients (BMI ≥ 30 kg/m^2^)

	Regression coefficient B	Standard error	*P*
TLR4 SNP rs4986790	−9.021	3.681	0.02
Age, y	0.574	0.133	<0.001
BMI, kg/m^2^	−0.849	0.385	0.03
HDL, mg/dl	−0.047	0.093	0.61
LDL, mg/dl	−0.012	0.028	0.67
Triglycerides, mg/dl	−0.004	0.008	0.63
Diabetes Mellitus, No versus Yes	2.023	3.060	0.50

dependent variable: systolic blood pressure, *BMI* body mass index, *LDL* low-density lipoprotein, *HDL* high-density lipoprotein, *TG* triglycerides

## Discussion

There is rising evidence that the innate immune system and in particular TLR4 and its ligands are involved in blood pressure modulation [18–20]. In diet-induced obesity there are numerous TLR4 ligands present [13, 21]. We addressed the question whether obesity-associated hypertension is reduced in TLR4 SNP rs4986790 cases [15]. Therefore we investigated a cohort of patients undergoing coronary angiography. We demonstrated that in control patients the BMI increase resulted in increased SBP, PP and hypertension. By contrast TLR4 SNP rs4986790 cases had no increase of SBP, PP and hypertension with BMI.

Strong evidence links TLR4-mediated inflammation in conditions such as obesity, insulin-resistance and diabetes [22–24]. In context with western diet saturated fatty acids, representing endogenous ligands of TLR4 are incorporated and cause inflammatory changes [25, 26]. Moreover, Western Diet alters the gut microbiota composition and increases intestinal permeability for the TLR4 ligand LPS. This process is called metabolic endotoxemia [27]. Thus, diet-induced obesity reflects a source of TLR4 ligands. These different sources of TLR4 ligands can directly act on vascular level as different types of arteries contain TLR4 [28].

In this study the known TLR4 SNP rs4986790 (Asp299Gly) was investigated [15]. This TLR4 SNP has been evidenced to reveal reduced reagibility upon TLR4 ligands [14]. The common missense mutation Asp299Gly affects the extracellular domain of the TLR4 receptor. This causes a blunted response to LPS exposition in humans [14]. Based on our hypothesis that TLR4 ligands are involved in obesity associated hypertension we expected a reduced blood pressure increase in obese patients with the TLR4 SNP rs4986790. Other studies, which deal with functional polymorphisms in toll-like receptor 4 could show on the one hand a worse outcome in acute ischemic stroke patients and were associated with myocardial infarction [29, 30]. On the other side the minor allele of the TLR4 SNP, rs1927911 was associated with a lower risk of myocardial infarction and the TLR 4 SNP rs4986790 showed no association with myocardial infarction [15, 29].

Our major finding was that TLR4 SNP rs4986790 cases were protected against the blood pressure increase across BMI quartiles. General characteristics were comparable between cases and controls and the Hardy–Weinberg equilibrium was comparable between BMI quartiles. Therefore the data were comparable between cases and controls. The blood pressure effect was in particular present in the obese cases as compared to the obese controls. This was not explained by intensified antihypertensive treatment or history of myocardial infarction. The regression analysis showed for obese patients that the TLR4 SNP rs4986790 remained a significant predictor of blood pressure even after multiple adjustments. The limitation of this study was that we restricted our analysis to one polymorphism. We did not perform a genome wide association study (GWAS). Additionally we did not measure the amount of TLR4 ligands in our patients.

## Conclusions

In summary our results suggest that in obesity associated hypertension TLR4 SNP rs4986790 cases present a lower SBP, pulse pressure and less hypertension. This finding needs to be confirmed in GWAS and functional studies need to be performed to address the role of TLR4 signaling in obesity associated hypertension.

## Abbreviations

TLR4: Toll-Like receptor 4
BMI: Body mass index
SNP: Single nucleotide polymorphism
LPS: Lipopolysaccharide
PCR: Polymerase chain reaction
Q: Quartile
HWE: Hardy-Weinberg equilibrium
LDL: Low-density lipoprotein
HDL: High-density lipoprotein
TG: Triglycerides
SBP: Systolic blood pressure
DBP: Diastolic blood pressure
PP: pulse pressure
HR: Heart rate
MI: Myocardial infarction
